# Sex difference in the associations among risk factors with depression in a large Taiwanese population study

**DOI:** 10.3389/fpubh.2023.1070827

**Published:** 2023-03-16

**Authors:** Hsin Tseng, Jia-In Lee, Jiun-Hung Geng, Szu-Chia Chen

**Affiliations:** ^1^Department of Post Baccalaureate Medicine, Kaohsiung Medical University, Kaohsiung, Taiwan; ^2^Department of Psychiatry, Kaohsiung Medical University Hospital, Kaohsiung Medical University, Kaohsiung, Taiwan; ^3^Department of Urology, Kaohsiung Municipal Siaogang Hospital, Kaohsiung Medical University, Kaohsiung, Taiwan; ^4^Department of Urology, Kaohsiung Medical University Hospital, Kaohsiung Medical University, Kaohsiung, Taiwan; ^5^Department of Internal Medicine, Kaohsiung Municipal Siaogang Hospital, Kaohsiung Medical University, Kaohsiung, Taiwan; ^6^Division of Nephrology, Department of Internal Medicine, Kaohsiung Medical University Hospital, Kaohsiung Medical University, Kaohsiung, Taiwan; ^7^Faculty of Medicine, College of Medicine, Kaohsiung Medical University, Kaohsiung, Taiwan; ^8^Research Center for Precision Environmental Medicine, Kaohsiung Medical University, Kaohsiung, Taiwan

**Keywords:** depression, sex difference, Taiwan Biobank, risk factors, interaction

## Abstract

**Background:**

Depression is a common psychiatric health issue affecting an estimated 5% of adults worldwide, and it can lead to disability and increased economic burden. Consequently, identifying the factors associated with depression as early as possible is a vital issue. The aim of this study was to explore these associations in a large cohort of 121,601 Taiwanese participants in the Taiwan Biobank, and also to identify sex differences in the associations.

**Methods:**

The study cohort included 77,902 women and 43,699 men (mean age, 49.9 ± 11.0 years), who were further classified into those with depression (*n* = 4,362; 3.6%) and those without depression (*n* = 117,239; 96.4%).

**Results:**

The results of multivariable analysis showed that female sex (vs. male sex; odds ratio = 2.578; 95% confidence interval = 2.319–2.866; *p* < 0.001) was significantly associated with depression. Older age, diabetes mellitus (DM), hypertension, low systolic blood pressure (SBP), smoking history, living alone, low glycated hemoglobin (HbA1c), high triglycerides, and low uric acid were significantly associated with depression in the men. In the women, older age, DM, hypertension, low SBP, smoking history, alcohol history, education level of middle and high school (vs. lower than elementary school), living alone, high body mass index (BMI), menopause, low HbA1c, high triglycerides, high total cholesterol, low estimated glomerular filtration rate (eGFR), and low uric acid were significantly associated with depression. Further, there were significant interactions between sex and DM (*p* = 0.047), smoking history (*p* < 0.001), alcohol use (*p* < 0.001), BMI (*p* = 0.022), triglyceride (*p* = 0.033), eGFR (*p* = 0.001), and uric acid (*p* = 0.004) on depression.

**Conclusion:**

In conclusion, our results showed sex differences in depression, and the women were significantly associated with depression compared to men. Furthermore, we also found sex differences among the risk factors associated with depression.

## Introduction

Depression is a common psychiatric health issue affecting an estimated 5% of adults worldwide, and it can lead to disability and increased economic burden (1). Although the reported lifetime prevalence in Taiwan is about 1.2%, this rate may be underestimated due to the culture of low help-seeking behavior (2). Demographic risk factors including younger age, female gender, lower household income, marital status of widowed, separated, or divorced, and comorbid psychiatric disorders are associated with an increased risk of depression (3). Depression has also been associated with multiple chronic medical diseases, and it is considered to be a significant contributor to suicide (1, 4, 5). Compared with the general population, people in Taiwan with depression have been found to have a shorter life expectancy and higher mortality rate (6). Therefore, identifying the potential risk factors for depression is a vital issue.

Sex differences have been reported in many diseases, including liver disease, cancer, and cardiovascular disease, and these differences manifest in clinical presentation, disease progression, and response to management (7). Associations between the disease and risk factors may also differ by sex. For example, the incidence of myocardial infarction has been reported to be three times higher in men than in women, whereas hypertension, smoking, and diabetes mellitus (DM) are associated with a greater relative risk in women than in men (8). Epidemiological studies have also revealed that the lifetime prevalence of depression is two times higher in women compared to men (3). These differences may be due to genetic factors, hormone modulation, stress response, or structural sex inequality (9). For depression, the relationship with risk factors is considered to be bidirectional. On one hand, depression may lead to unhealthy behaviors such as smoking and alcohol consumption, which then elevate the possibility of developing chronic diseases. On the other hand, poor physical health conditions may cause depression due to common pathogenesis or the increased need for psychological support (4, 5). Although previous studies have established bidirectional relationships between depression and comorbid conditions, whether sex differences also influence the risk factors is unclear. Therefore, we conducted this study to explore sex differences in the associations among risk factors with depression in a large cohort of Taiwanese participants.

## Materials and methods

### Taiwan Biobank

Due to societal aging in Taiwan, the Ministry of Health and Welfare announced a policy to counter chronic diseases through health promotions, and consequently launched the TWB. Volunteers are enrolled in the TWB, with the inclusion criteria of an age between 30 and 70 years and no prior diagnosis of cancer. In this study we used data from 121,601 participants in the TWB, including lifestyle habits, medical and genetic information as detailed in the following section (10, 11).

### Medical data, demographics, lifestyle habits, and laboratory data

All enrollees in the TWB are interviewed to obtain personal information on their age, sex, lifestyle factors (i.e., exercise), educational status, living alone status, and medical history (i.e., DM and hypertension). In this study, we defined regular exercise as exercising for at least 30 min three times or more in 1 week.

Body height and weight were also recorded for each enrollee, along with the body mass index (BMI) (kg/m^2^). Blood samples were also drawn from each enrollee, from which glycated hemoglobin (HbA1c), hemoglobin, triglycerides, total cholesterol, and uric acid were measured. Estimated glomerular filtration rate (eGFR) was also recorded using the MDRD Study equation [186 × serum creatinine^−1.154^ × Age^−0.203^ × 0.742 (if female) × 1.212 (if black patient)] (12). DM was defined as self-reported, fasting glucose level ≥126 mg/dL or HbA1c ≥ 6.5%. Participants had past history of hypertension (self-reported), and whose systolic blood pressure was >140 mmHg and diastolic blood pressure was >90 mmHg were defined to have hypertension.

### Depression groups

The participants were classified into two groups according to whether or not they had ever had depression. Those who answered “Yes” to the question “Have you ever had depression?” were classified into the depression group, and those who answered “No” were classified into the without depression group.

### Ethical considerations

The Institutional Review Board of Kaohsiung Medical University Hospital approved this study (KMUHIRB-E(I)-20210058). Ethical approval for the TWB was granted by the IRB on Biomedical Science Research, Academia Sinica, Taiwan and the Ethics and Governance Council of the TWB. In addition, the study was conducted in accordance with the Declaration of Helsinki, and all of the participants gave written informed consent.

### Statistical analysis

Continuous variables are presented as mean (±SD), with differences analyzed using the independent *t*-test. Categorical variables are presented as percentage, with differences analyzed using the chi-square test. Correlations among risk factors with depression were analyzed with multivariable logistic regression analyses. An interaction *p* in logistic analysis was identified using the following formula: Model disease (*y*) = *x*1 + *x*2 + *x*1^*^x2 + covariates *x*1^*^x2, where y = depression; *x*1 = sex; *x*2 = each risk factor; covariates = age, sex, DM, hypertension, systolic and diastolic blood pressures, smoking and alcohol history, regular exercise habit, education status, living alone, BMI, HbA1c, hemoglobin, triglycerides, total cholesterol, eGFR and uric acid. Results were considered significant at *p* < 0.05. Statistical analysis was performed using SPSS for Windows (v26, SPSS Inc. Armonk, NY, USA).

## Results

The enrolled participants (*n* = 121,601; mean age 49.9 ± 11.0 years; 77,902 females; 43,699 males) were divided into two groups according to those with depression (*n* = 4,362; 3.6%) and without depression (*n* = 117,239; 96.4%).

### Comparisons of clinical characteristics between the two depression groups

Compared to the without depression group, the with depression group were older, had a higher proportion of females, higher prevalence of DM, hypertension, living alone, menopause status, and smoking, regularly exercised more. In addition, the with depression group had lower systolic and diastolic blood pressures, lower prevalence of educational status higher than college, higher levels of HbA1c, triglycerides and total cholesterol, and lower levels of hemoglobin, eGFR, and uric acid (Table 1).

**Table 1:** Comparison of clinical characteristics among participants without or with depression.

**Characteristics**	**Depression (–) (*n* = 117,239)**	**Depression (+) (*n* = 4,362)**	***p***
Age (year)	49.8 ± 11.0	51.3 ± 10.4	< 0.001
Female (%)	63.6	76.1	< 0.001
DM (%)	5.1	7.8	< 0.001
Hypertension (%)	12.1	16.0	< 0.001
Systolic BP (mmHg)	120.5 ± 18.7	119.2 ± 18.2	< 0.001
Diastolic BP (mmHg)	73.8 ± 11.4	73.0 ± 11.2	< 0.001
Smoking history (%)	27.1	31.6	< 0.001
Alcohol history (%)	8.5	9.0	0.235
Regular exercise habits (%)	40.5	42.0	0.046
**Education status**			< 0.001
Lower than elementary school (%)	5.3	5.8	
Middle and high school (%)	36.5	43.9	
Higher than college (%)	58.2	50.3	
Living alone (%)	7.9	13.9	< 0.001
BMI (kg/m^2^)	24.2 ± 3.8	24.2 ± 4.1	0.437
Menopause in female (%)	45.5	52.3	< 0.001
**Laboratory parameters**			
HbA1c (%)	5.76 ± 0.80	5.79 ± 0.82	0.034
Hemoglobin (g/dL)	13.8 ± 1.6	13.5 ± 1.5	< 0.001
Triglyceride (mg/dL)	115.5 ± 94.3	119.9 ± 85.5	0.002
Total cholesterol (mg/dL)	195.5 ± 35.8	198.4 ± 35.7	< 0.001
eGFR (mL/min/1.73 m^2^)	103.3 ± 23.9	102.5 ± 24.2	0.029
Uric acid (mg/dL)	5.4 ± 1.4	5.2 ± 1.4	< 0.001

DM, diabetes mellitus; BP, blood pressure; BMI, body mass index; HbA1c, glycated hemoglobin; eGFR, estimated glomerular filtration rate.

### Determinants of depression

The factors associated with depression in multivariable logistic regression analysis for the whole study cohort (*n* = 121,601) are shown in Table 2. This model adjusted age, sex, DM, hypertension, systolic and diastolic blood pressures, smoking and alcohol history, regular exercise, educational status, living alone, BMI, HbA1c, hemoglobin, triglycerides, total cholesterol, eGFR and uric acid. After analysis, older age, female (vs. male; odds ratio [OR] = 2.578; 95% confidence interval [CI] = 2.319–2.866; *p* < 0.001), DM, hypertension, low systolic blood pressure, high diastolic blood pressure, smoking history, educational level of middle and high school (vs. lower than elementary school), living alone, high BMI, low HbA1c, high triglycerides, high total cholesterol, low eGFR, and low uric acid were significantly associated with depression.

**Table 2:** Determinants for depression using multivariable logistic regression analysis in all study participants (*n* = 121,601).

**Parameters**	**Depression**		
	**Multivariable**		
	**OR**	**95% CI**	***p***
Age (per 1 year)	1.012	1.008–1.016	< 0.001
Female (vs. male)	2.578	2.319–2.866	< 0.001
DM	1.669	1.447–1.925	< 0.001
Hypertension	1.399	1.272–1.539	< 0.001
Systolic BP (per 1 mmHg)	0.991	0.989–0.994	< 0.001
Diastolic BP (per 1 mmHg)	1.005	1.001–1.010	0.022
Smoking history	1.978	1.826–2.142	< 0.001
Alcohol history	1.106	0.985–1.241	0.087
Regular exercise habits	1.013	0.949–1.082	0.703
**Education status**			
Lower than elementary school	Reference		
Middle and high school	1.236	1.076–1.419	0.003
Higher than college	1.063	0.922–1.225	0.403
Living alone	1.734	1.586–1.897	< 0.001
BMI (per 1 kg/m^2^)	1.010	1.000–1.019	0.041
**Laboratory parameters**			
HbA1c (per 1%)	0.917	0.874–0.963	0.001
Hemoglobin (per 1 g/dL)	1.002	0.977–1.027	0.899
Triglyceride (per 10 mg/dL)	1.005	1.003–1.008	< 0.001
Total cholesterol (per 1 mg/dL)	1.001	1.000–1.002	0.006
eGFR (per 1 mL/min/1.73 m^2^)	0.996	0.995–0.998	< 0.001
Uric acid (per 1 mg/dL)	0.944	0.917–0.972	< 0.001

Values expressed as odds ratio (OR) and 95% confidence interval (CI). Abbreviations are the same as in Table 1.

Adjusted for age, sex, diabetes, hypertension, systolic and diastolic blood pressures, smoking and alcohol history, regular exercise habit, education status, living alone, BMI, HbA1c, hemoglobin, triglyceride, total cholesterol, eGFR and uric acid.

### Determinants of depression by sex

The factors associated with depression by sex in multivariable logistic regression analysis are shown in Table 3. In the male participants (*n* = 43,699), older age, DM (OR = 1.524; 95% CI = 1.179–1.971; *p* = 0.001), hypertension, low systolic blood pressure, smoking history (OR = 1.395; 95% CI = 1.218–1.597; *p* < 0.001), living alone, low HbA1c, high triglycerides, and low uric acid (per 1 mg/dL; OR = 0.918; 95% CI = 0.873–0.965; *p* = 0.001) were significantly associated with depression. In the female participants (*n* = 77,902), older age, DM (OR = 1.753; 95% CI = 1.476–2.082; *p* = 0.047), hypertension, low systolic blood pressure, smoking history (OR = 2.323; 95% CI = 2.115–2.551; *p* < 0.001), alcohol history (OR = 1.348; 95% CI = 1.141–1.598; *p* < 0.001), educational level of middle and high school (vs. lower than elementary school; OR = 1.231; 95% CI = 1.060–1.428; *p* = 0.006), living alone, high BMI, menopause, low HbA1c, high triglycerides, high total cholesterol, low eGFR (per 1 ml/min /1.73 m^2^; OR = 0.996; 95% CI = 0.994–0.997; *p* < 0.001), and low uric acid (per 1 mg/dL; OR = 0.951; 95% CI = 0.917–0.986; *p* = 0.006) were significantly associated with depression.

**Table 3:** Association of risk factors with depression using multivariable logistic regression analysis in different sex.

**Parameters**	**Male (*****n*** = **43,699)**			**Female (*****n*** = **77,902)**			**Interaction *p***
	**Multivariable** ^*^			**Multivariable** ^#^			
	**OR**	**95% CI**	***p***	**OR**	**95% CI**	***p***	
Age (per 1 year)	1.015	1.008–1.023	< 0.001	1.006	1.001–1.012	0.031	0.265
DM	1.524	1.179–1.971	0.001	1.753	1.476–2.082	< 0.001	0.047
Hypertension	1.403	1.187–1.659	< 0.001	1.420	1.263–1.595	< 0.001	0.084
Systolic BP (per 1 mmHg)	0.989	0.983–0.995	< 0.001	0.992	0.989–0.995	< 0.001	0.082
Diastolic BP (per 1 mmHg)	1.008	0.999–1.016	0.091	1.004	0.999–1.010	0.089	0.295
Smoking history	1.395	1.218–1.597	< 0.001	2.323	2.115–2.551	< 0.001	< 0.001
Alcohol history	1.006	0.859–1.178	0.941	1.348	1.141–1.598	< 0.001	< 0.001
Regular exercise habits	0.997	0.874–1.138	0.963	1.013	0.939–1.093	0.735	0.859
**Education status**							
Lower than elementary school	Reference			Reference			
Middle and high school	1.384	0.939–2.039	0.101	1.231	1.060–1.428	0.006	0.524
Higher than college	1.283	0.873–1.888	0.205	1.045	0.895–1.221	0.576	0.123
Living alone	1.876	1.541–2.283	< 0.001	1.661	1.501–1.838	< 0.001	0.374
BMI (per 1 kg/m^2^)	1.003	0.983–1.023	0.781	1.011	1.000–1.020	0.044	0.022
Menopause in female	–	–		1.189	1.070–1.321	0.001	
**Laboratory parameters**							
HbA1c (per 1%)	0.899	0.824–0.980	0.016	0.921	0.868–0.978	0.007	0.063
Hemoglobin (per 1 g/dL)	1.020	0.967–1.077	0.462	0.986	0.958–1.015	0.350	0.688
Triglyceride (per 10 mg/dL)	1.005	1.001–1.009	0.021	1.007	1.003–1.011	0.001	0.033
Total cholesterol (per 1 mg/dL)	1.001	0.999–1.003	0.317	1.001	1.000–1.002	0.024	0.422
eGFR (per 1 mL/min/1.73 m^2^)	1.001	0.997–1.004	0.738	0.996	0.994–0.997	< 0.001	0.001
Uric acid (per 1 mg/dL)	0.918	0.873–0.965	0.001	0.951	0.917–0.986	0.006	0.004

Values expressed as odds ratio (OR) and 95% confidence interval (CI).

* Adjusted for age, sex, diabetes, hypertension, systolic and diastolic blood pressures, smoking and alcohol history, regular exercise habit, education status, living alone, BMI, HbA1c, hemoglobin, triglyceride, total cholesterol, eGFR and uric acid.

# Adjusted for age, sex, diabetes, hypertension, systolic and diastolic blood pressures, smoking and alcohol history, regular exercise habit, education status, living alone, BMI, HbA1c, hemoglobin, triglyceride, total cholesterol, eGFR, uric acid and menopause.

### Interactions among risk factors and sex on depression

There were significant interactions between sex and DM (*p* = 0.047), smoking history (*p* < 0.001), alcohol use (*p* < 0.001), BMI (*p* = 0.022), eGFR (*p* = 0.001), and uric acid (*p* = 0.004) on depression (Table 3).

## Discussion

The results of this large-scale study showed that the female participants were significantly associated with depression compared to the male participants. Furthermore, we found sex differences in the associations among the risk factors. There were significant interactions between sex and DM, smoking history, alcohol use, BMI, eGFR, and uric acid on depression.

A main finding of this study is that the female participants had a higher rate of depression compared to the male participants. The prevalence of depression has been shown to increase significantly during puberty, with a greater increase in girls (13). This difference remains relatively stable into adulthood, and even after menopause in women (14). The reason for the higher prevalence of depression in women cannot be explained by a single mechanism, and is likely to be due to risk factors including genetic factors, sex hormones, and stress (9, 15). Considering the effect of genetic factors, although depression is a familial disorder with heritability ranging around 30~40% (16), genetic influences specifically in females have yet to be definitively concluded. Some studies have reported that genetic factors have a considerable impact on females (17), whereas other studies have reported that the impact is greater in males (18). Thus, more research is required not only on direct genetic factors, but also on the influence of confounding factors such as environmental factors, demographic characteristics, study measurements, and so on (19). Sex hormones are also considered to be an important risk factor for depression in women (20). Women pass through different phases during their lifetime, including puberty, premenstrual dysphoric disorder and mood swings before menstruation, postpartum depression, and depression during perimenopause and menopause, all of which are related to fluctuations in sex hormones (21, 22). In addition, stress is associated with the risk of the first onset, recurrence, and exacerbation of depression (23). Previous studies have reported that adolescent girls and women may encounter sexual abuse and domestic violence, experience greater interpersonal stress, housing problems, and the burden of taking care of others (23, 24). Thus, the effects of sex hormones, stress, and genetic factors may explain the higher risk of depression in women than in men.

Another main finding of the present study is the significant interaction between sex and DM on depression, and DM was more strongly associated with depression in the female participants than in the male participants. Previous studies have shown that patients with DM have an increased risk of developing depression, while people suffering from depression also have a higher chance of developing DM (25). Women with DM have been reported to have a more than twofold higher risk of being diagnosed with depression compared to women without DM, while the effect is significantly smaller in men (26). The mechanism for this disparity is still unclear, but it may be due to differences in risk, glucose tolerance, and insulin sensitivity between sexes. With regards to the risk of DM, men have been reported to have a lower age and BMI at the time of diagnosis, whereas women have been more related to obesity (27). In addition, obesity has been reported to have a stronger link with depression in women than in men, which is due to behavioral and psychosocial impairment and hypothalamic–pituitary–adrenal axis dysfunction, a stress regulation problem related to psychiatric disorders (27, 28). We also found that the female participants with high BMI had an increased risk of depression, whereas the risk was not significant in the male participants. In response to oral glucose tolerance tests, men often have impaired fasting glucose while women usually have impaired glucose tolerance (29). These differences may be associated with impairment in first- or second-phase insulin secretion, respectively, stimulated by glucose (30). Although insulin sensitivity and insulin secretion status are similar in men and women diagnosed with DM, the reduction in insulin sensitivity is greater in women than in men when the metabolic condition declines from normal to illness (27). Confounders of menopause including lower skeletal muscle mass, body fat distribution, and higher androgen activity and testosterone level, especially estrogen deficiency, have also been linked to elevated insulin resistance and the risk of DM in middle-aged women (31, 32). Taken together, these mechanisms may partly explain the relationship between DM and depression in women.

Another important finding of this study is the significant interaction between sex and alcohol and smoking history on depression, and the association was stronger in the female participants than in the male participants. Many studies have postulated a positive association between substance use and depressive symptoms in young adolescents, and that this association is more pronounced in girls (33, 34). In middle-aged and older adults, smoking has been associated with a 20% higher risk of developing depression, while those with depression have been reported to have 41 and 18% higher risks of starting to smoke and heavy drinking, respectively (35). In addition, any amount of alcohol consumption has been associated with a greater increase in depressive episodes in women than in men, especially with the synergistic effects of smoking (36). Possible mechanisms for the relationship between smoking and depression include the self-medication hypothesis, alternative hypothesis, bidirectional relationship, or there may be no relationship at all (37). Smoking may be a way to alleviate the symptoms of depression or keep individuals in a vulnerable state to environmental stress by regulating the hypothalamic–pituitary–adrenal axis (38, 39). It could also be a bidirectional relationship, or just a consequence of sharing common risk factors so that there is actually no direct relationship. With regards to the influence of alcohol use on depression, previous studies have suggested that a bidirectional and mutually reinforcing relationship may explain the correlation between alcohol use disorders and major depression (40). The association may be due to psychosocial impairment resulting from chronic heavy drinking that eventually elicits depressive symptoms or affects the release of neurotransmitters such as dopamine and gamma-aminobutyric acid receptors (41). Taken together, an increased risk of depression related to smoking and alcohol consumption has been reported in females of all ages due to the influence of psychosocial, biological, and environmental factors.

Another interesting finding of the present study is the negative correlation between eGFR and depression found in the female participants but not in the male participants. Depression has been associated with poor clinical outcomes of chronic kidney disease (CKD), including faster eGFR decline, early dialysis therapy initiation, death, or hospitalization (42, 43). In adults with normal kidney function, the presence of depressive symptoms has also been associated with a higher risk of rapid kidney function decline (44). The mechanism for this relationship is unclear, but it may be related to inflammation or stress-related physiological changes. Previous studies have reported that patients with kidney disease have higher levels of interleukin-6 (IL-6) and C-reactive protein, which are both considered to be related to the severity of depression due to increased production through various pro-inflammatory pathways and decreased clearance (45). On the other hand, higher levels of psychological stress have been shown to increase the progression of kidney function decline, which can also result in depression by affecting the immune and endocrine systems (46, 47). Sex differences have also been observed in the epidemiology of CKD, with a higher rate in women than in men. Women with CKD often suffer from a higher burden and stress, have a greater severity of symptoms, and handle the disease in a more emotional way, which can then predispose to the development of depression (48). However, further investigations are required to elucidate the underlying mechanism and the influence of depression on kidney function in people with normal kidney function.

We also noted that compared to lower than elementary school, educational level of middle and high school, not higher than college, were associated with depression. Studies have been revealed that education plays an important role in protection against depression with individuals receiving lower levels of education may have higher rates of depression. It was also indicated that adults with depression have lower educational aspirations and expectations and often their parents were also less educated (49). Education is often considered to influence health, not only depression, through mechanisms of economy, health behavior, social-psychology, and access to health care. Higher levels of educations provide better socioeconomic conditions, less unhealthy lifestyle, better coping skills of stressors and daily hassles, and better management of health problems (50). Interestingly, it was found that education has stronger health effects on women than men, especially on self-rated health (51). Taken together, education have an important relationship with depression; people with lower education level would have higher rate of experiencing depression.

The last important finding of this study is the significant interaction between sex and uric acid on depression, with low uric acid being more strongly associated with depression in the male participants than in the female participants. Previous studies have suggested an association between depression and lower levels of serum uric acid (52, 53). Meng et al. (53) reported that although the differences were small compared to normal controls, the change in serum uric acid was consistent between subtypes of depression. In addition, Black et al. (52) proposed that the lower levels may be related to a greater severity and longer duration of depressive symptoms. Uric acid is the end product of purine metabolism and also considered a strong peroxynitrite scavenger, and it plays a role in dealing with oxidative stress (54). Biological changes in purine metabolism and oxidative stress may be involved in the relationship with depression. The purinergic system has been linked to mood disorders through dysfunction driven by adenosine triphosphate and P2Y receptors, which regulate drive, cognition, appetite, sleep and mood (55). The excessive oxidative stress associated with depression can lead to increased consumption of uric acid as an antioxidant (56). Our results suggest that hyperactive purine degeneration with lower serum levels of inosine and guanosine and higher serum levels of xanthine may be associated with low uric acid in patients with depression (57). In addition, we found that low uric acid was more strongly associated with depression in the male participants than in the female participants. This disparity may be due to sex differences in oxidative stress, as women under chronic stress have been shown to have better antioxidative capacity, lower reactive oxygen species-induced damage, and estrogen-driven protection, resulting in lower consumption of uric acid (58, 59). Further studies on the sex-specific association between serum uric acid and depression are needed to elucidate the mechanism.

The key strengths of this research are that we included a large cohort of healthy community-dwelling participants, and the comprehensive control of confounding factors. However, several limitations should also be noted. As this study was cross-sectional it was not possible to determine how long each participant had depression, and consequently we could not conclude causal relationships between the risk factors and depression. Longitudinal studies are needed to investigate sex differences and incident depression. Second, the prevalence of depression in TWB is 3.6%, higher than previous reported 1.2% (2). However, data on the presence of depression were obtained from self-reported questionnaires, and depression was not verified by psychiatrist diagnosis, which may not be rigorous enough. In addition, it is possible that some participants took medications for hypertension, glucose, hyperuricemia and lipid control. However, data on such medications are not provided by the TWB. Therefore, we could not evaluate the effects of these medications on the association between laboratory data and depression. Another limitation is that we could not ascertain the severity of depression. Fifth, the enrolled participants were all ethnically Chinese, and thus caution should be taken when extending our results to other ethnicities. Finally, because the average age in the depression group is higher than non-depression group, cohort effect could not be excluded.

In conclusion, our results showed sex differences in the incidence of depression, and the female participants were significantly associated with depression compared to the male participants. Furthermore, we found sex differences in the associations among the risk factors with depression in this a large study of Taiwanese participants.

## Data availability statement

The raw data supporting the conclusions of this article will be made available by the authors, without undue reservation.

## Ethics statement

The studies involving human participants were reviewed and approved by the Institutional Review Board of Kaohsiung Medical University Hospital approved this study (KMUHIRB-E(I)-20210058). The patients/participants provided their written informed consent to participate in this study.

## Author contributions

Conceptualization, methodology, validation, formal analysis, writing—review and editing, and supervision: HT, J-IL, J-HG, and S-CC. Software and investigation, resources, project administration, funding acquisition, and visualization: S-CC. Data curation: J-IL, J-HG, and S-CC. Writing—original draft preparation: HT, J-IL, and S-CC. All authors have read and agreed to the published version of the manuscript.
